# Temperature Effect on the Mechanical Properties of Electrospun PU Nanofibers

**DOI:** 10.1186/s11671-018-2801-1

**Published:** 2018-11-29

**Authors:** Ji Zhou, Qing Cai, Xing Liu, Yanhuai Ding, Fu Xu

**Affiliations:** 1 College of Civil and Environmental Engineering, Hunan University of Science and Engineering, Yongzhou, 425006 Hunan China; 2 College of Civil Engineering and Mechanics, Xiangtan University, Xiangtan, 411105 Hunan China

**Keywords:** PU nanofiber, Mechanical property, AFM, Young’s modulus, Three-point bending test

## Abstract

Polyurethane (PU) nanofibers were prepared from electrospun method. Atomic force microscopy (AFM) was employed to characterize the mechanical properties of electrospun PU nanofibers. The impact of temperature on the mechanical behavior of PU nanofibers was studied using three-point bending test based on AFM. A Young’s modulus of ~ 25 GPa was obtained for PU nanofibers with diameter at ~ 150 nm at room temperature. With decrease in nanofiber’s diameter, the increasing Young’s modulus can be due to the surface tension effect. The Young’s modulus of the PU nanofiber decreased linearly while the fibrous morphology was maintained with the increase of temperature.

## Background

One-dimensional (1D) nanomaterials have been intensively studied due to their unique properties and intriguing applications in many areas [1–3]. Many synthetic and fabricating methods have already been explored to generate 1D nanostructures in the form of fibers, wires, rods, and tubes from various materials [4, 5]. However, their usefulness is limited by combinations of restricted material ranges, cost, and production rate. Unlike other methods for generating 1D nanostructures, electrospinning has an advantage with its relative low cost and high production rate, which is similar to the commercial processes for producing microscale fibers except for the use of electrostatic repulsions to continuously reduce the diameter of a viscoelastic jet [6, 7].

Polyurethane (PU) is composed of soft and hard segments connected by a urethane linkage, in which the soft segments impart flexibility, whereas the hard segments provide the rigidity and strength [8, 9]. PU materials have been widely used in industry since its hardness can be easily modulated by changing the hard-segment in the structure [10]. Electrospun PU nanofibers have a wide variety of potential applications in high-performance air filters, protective textiles, wound dressing films, and sensors [11, 12]. Understanding the mechanical properties is essential for the application and function of nanomaterials [13]. However, too little attention has been paid to the study of mechanical properties of electrospun nanofibers due to the difficulties in making a nanoscale test. In the past decade, atomic force microscopy (AFM) was employed to characterize the mechanical properties of 1D nanostructure in a straightforward way [14–16]. A facile AFM-based three-point bending test has been designed to measure the Young’s modulus of a single nanofiber, which involves clamping the 1D nanostructure across a trench by the self-adhesion between the sample and substrate. The midpoint of the suspended 1D nanostructure is subjected to a force applied by the AFM tip, and then, the corresponding deflection at the midpoint is recorded and used to calculate the Young’s modulus. Here, PU nanofibers were prepared from electrospun method. And then three-point bending test was employed to study the effect of temperature on the Young’s modulus of PU nanofibers.

## Methods

### Material Preparation

N, N-dimethylformamide (DMF) and tetrahydrofuran (THF) were purchased from Tianjin Hengxing Chemical Reagent Co., Ltd. Polyurethane elastomer (Elastollan® 1180A10) was obtained from BASF. PU was dissolved in the mixture of DMF and THF with a volume ratio of 1:1. The solution was sealed at room temperature for more than 12 h with intensive stirring. A commercially available electrospinning set-up (Beijing Ucalery Technology Development Co., Ltd., China) was used for the fabrication of electrospun PU nanofibers. The distance between the nozzle and a grounded collector was adjusted to 13 cm. A high voltage of 9–10 kV was applied to generate a polymer jet. The resulting fibers were collected on a rotating mandrel, left in vacuum conditions overnight to eliminate solvent residues and then kept in a desiccator for further experimentation.

### Physical Characterization and Testing Method

The microstructure and morphology of the as-prepared PU nanofibers were characterized by scanning electron microscopy (SEM, JSM-6610LV, Japan). Thermogravimetric differential scanning calorimetry (TG/DSC) analysis was carried out with a DSC–TGA (SDT Q600, TA Instruments) under argon atmosphere. The macroscopic elastic modulus of electrospun PU membrane was measured by universal testing machine (Instron 5943, USA). The nanomechanical properties of nanofibers were tested by using Multimode 8 AFM (Bruker Nano Inc., USA). First, electrospun PU nanofibers were deposited by using a Si template as collector (purchased from Suzhou RDMICRO Co., Ltd.). The nanofibers suspended on the groove were submitted to AFM test. The width and depth of the groove on the substrate are 2 and 3 μm. The probe is simplified as a sphere with a diameter of 50 nm. The spring constant of the cantilever was measured by thermal tune method. Sensitivity of the cantilever, as the cantilever deflection signal vs. the applied voltage, was calibrated on a sapphire surface. Force curves were recorded to calculate the elastic modulus of a single nanofiber. Each experiment was repeated 5 times, and the results were averaged (arithmetic mean). Finite element simulation was performed to evaluate the degree of tip penetration into the nanofiber surface. The simulation model was established in commercial software package (ANSYS 15.0). The materials of nanofiber, probe, and the substrate are all considered as elastic linear isotropic solids [17].

## Results and Discussion

**Fig. 1:**
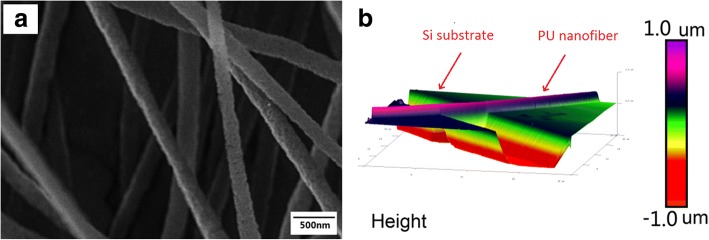
SEM (**a**) and AFM (**b**) images of electrospun PU nanofibers

The morphological features of the electrospun PU nanofibers were characterized by SEM and AFM. As shown in Fig. 1a, electrospun PU film is composed of randomly oriented nanofibers with the diameter ranged from hundreds of nanometers to several micrometers. AFM image in Fig. 1b demonstrates that the PU nanofibers are uniform in the lateral section. The diameter of the nanofiber measured by AFM was ~ 300 nm.

**Fig. 2:**
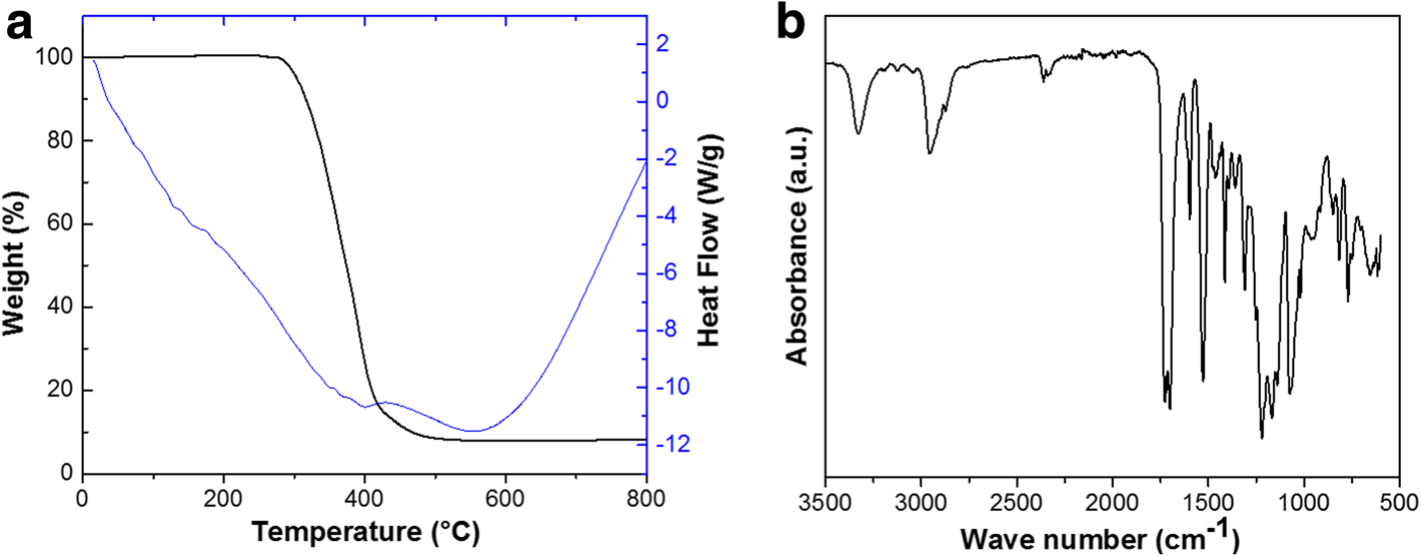
TG/DSC curves (**a**) and FTIR spectrum (**b**) of electrospun PU nanofibers

Figure 2a shows the TG/DSC curves of electrospun PU nanofibers in argon at a heating rate of 10 °C/min. It is obvious that the thermal degradation of PU nanofibers in argon show a two-stage process. Small amount of weight loss can be observed between 100 and 200 °C, indicating the evaporation of water and some small molecules products in this stage. The weight loss observed at 300 °C is related to the decomposition of the polymer [18, 19]. Nevertheless, only a small endothermic peak is shown in argon, corresponding to the main weight loss stage. As shown in Fig. 2b, the FTIR spectrum of electrospun PU has characteristic absorption bands at 3320, 2960, 1710, 1530, 1220, 1110, and 777 cm^−1^, which represents *υ*_(N–H)_, *υ*_(C–H)_, *υ*_(C–O)_, *υ*_(C–C)_, *υ*_(C–C)_, *υ*_(C–O)_, and *υ*_(C–H)_, respectively [18].

**Fig. 3:**
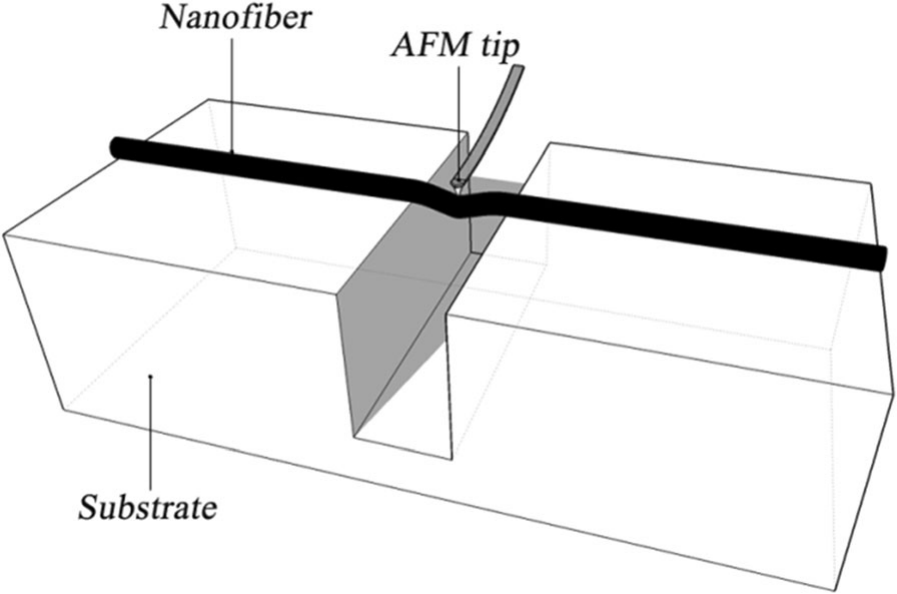
Scheme of the three-point bending test

In the three-point bending test, PU nanofibers were deposited on the surface of Si wafer as shown in Fig. 3. The three-point beam bending theory for a beam with two ends fixed has been widely used to calculate the Young’s modulus of a nanofiber as follows:

1 $$ E={FL}^3/192 dI $$

where *F* is the force applied at the midpoint, *L* is the suspended length of the nanofiber, *d* is the deflection of the nanofiber at the midpoint, and *I* is the section inertia moment (*I* = π*r*^4^/4, where *r* is the radius of the fiber). The following assumptions should be met to calculate the Young’s modulus [20]: (i) the two ends of the fiber are fixed, (ii) *L* is much larger than the *r*, and (iii) *d* is very small. In our work, no relative slippage between the nanofiber and substrate was observed in the test. It has been concluded that the calculation error can be controlled in 8% with the *L*/*r* greater than 10 in the previous work [17]. Thus, these assumptions can be satisfied during the three-point bending test. The simulated results from finite element method indicate that the depth of tip penetration is below 10% of the deformation of nanofibers. So the elastic modulus is calculated based on the assumption that the surface deformation can be ignored.

**Fig. 4:**
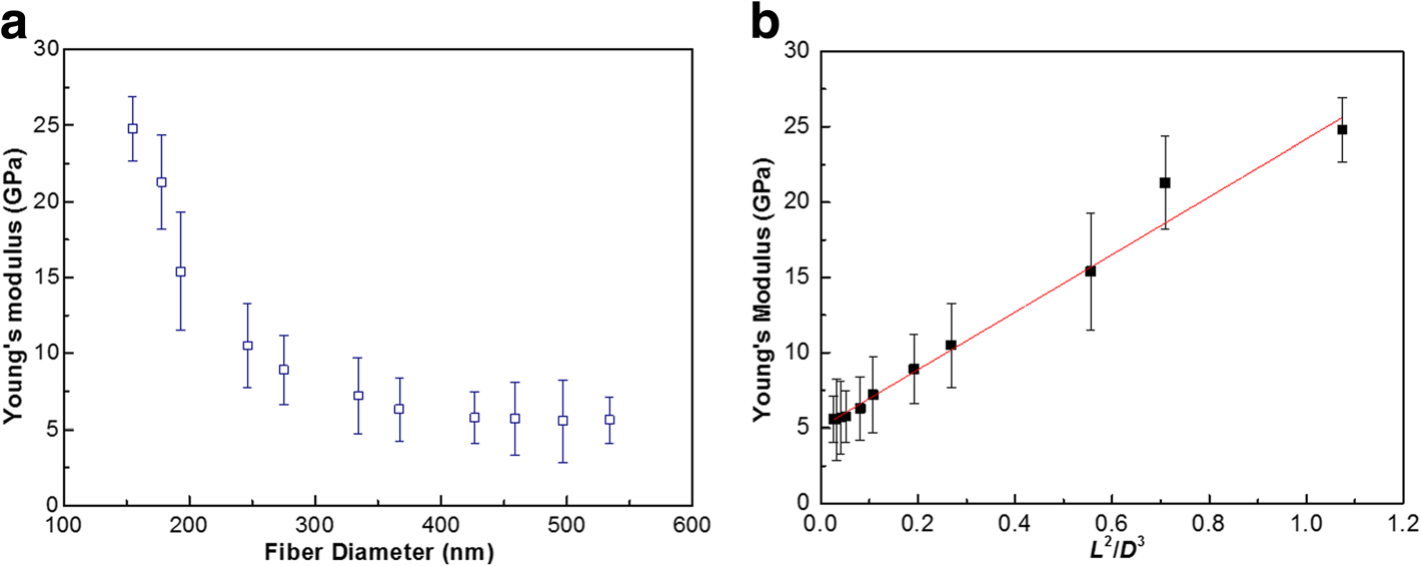
**a** A plot of Young’s modulus against the diameter of PU nanofibers. **b** The surface tension effect on the mechanical properties of PU nanofibers

Figure 4a shows results of the three-point bend test as a plot of Young’s modulus against the diameter of the PU nanofibers. Young’s modulus of a single PU nanofiber is indicated in the figure. Young’s modulus of PU nanofibers exhibits diameter dependence. The modulus value increases as the diameter decreases below a certain size of about 300 nm. A high Young’s modulus of ~ 25 GPa can be obtained with a diameter of 150 nm, while the Young’s modulus decreases to ~ 5 GPa with the diameter greater than 300 nm. In the recent works, the Young’s moduli of polymer nanofibers such as nylon 6, Poly(ε-caprolactone), cellulose, and polyvinyl alcohol measured by AFM-based three-point bending test was in the range from several GPa to tens of GPa [21–23]. The Young’s modulus of PU nanofibers measured in this work was also in the range mentioned above. The macroscopic mechanical properties of the electrospun PU membrane were also measured. A Young’s modulus of 0.9 MPa can be obtained, which can be ascribed to the high porosity of the electrospun membrane.

As reported in the previous work [24], the observed increase in the Young’s modulus with decreasing diameter is essentially due to surface tension effects. Taking into account the surface effect, the apparent Young’s modulus can be expressed as:

2 $$ E={E}_0+\frac{8\gamma \left(1-\nu \right)}{5}\frac{L^2}{D^3} $$

where *E*_0_, *γ*, and *υ* is the Young’s modulus, surface tension, and Poisson’s ratio of the bulk materials, respectively. *D* is the diameter of the nanofiber. As shown in Fig. 4b, linear regression allows the determination of the elastic modulus and surface tension. Thus, the intrinsic Young’s modulus of PU nanofiber is about ~ 5.0 GPa, which is much greater than that of the bulk materials. The reason for this is that the molecular chains were oriented within the electrospun fibers during the electrospinning process [25].

**Fig. 5:**
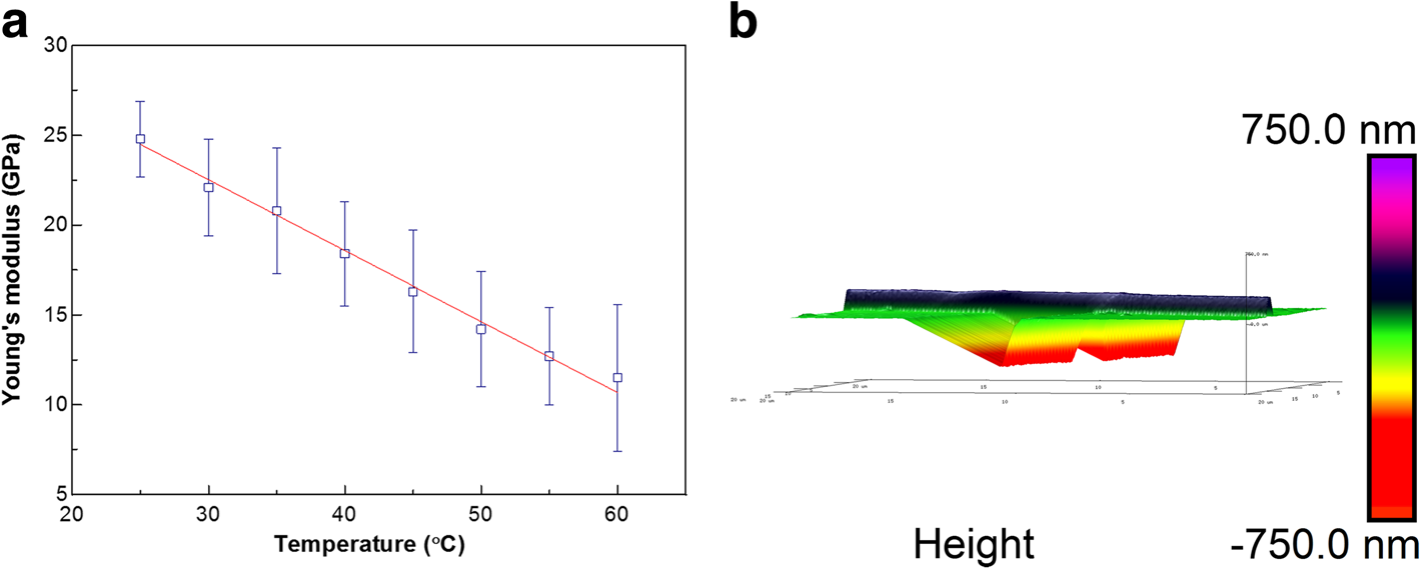
**a** The temperature effect on the Young’s modulus of a single PU nanofiber. **b** The morphology of a single PU nanofiber at 60 °C

The temperature effect on the Young’s modulus of a single PU nanofiber is presented in Fig. 5a. For a single PU nanofiber with a diameter of 155 nm, the Young’s modulus decreases linearly with the increase in temperature in the range of 25 °C~ 60 °C. However, AFM images in Fig. 5b confirm that the fibrous morphology of the PU nanofiber is completely maintained with the temperature being increased to 60 °C. Lateral section profile indicates that the diameter of the measured PU nanofiber increases slightly from 200 to 214 nm. We can conclude that PU nanofiber possesses high dimension stability at relatively low temperatures. Besides, the linear relationship between the Young’s modulus and temperature suggests the potential application of electrospun PU nanofibers in the field of nanodevices and nanosensors.

**Fig. 6:**
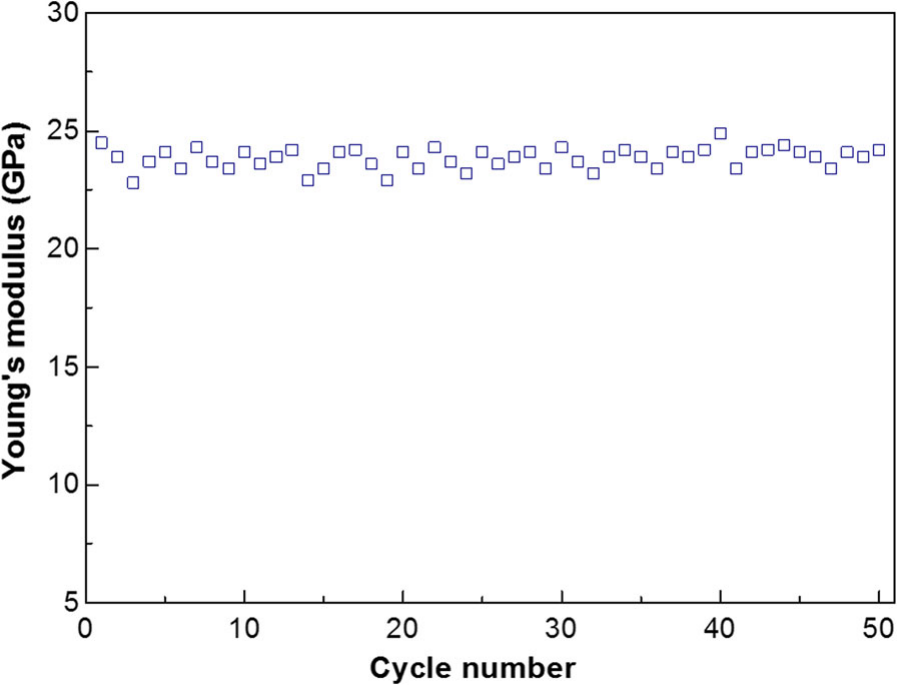
The degradation of mechanical properties of a single PU nanofiber

The degradation of mechanical properties of a single PU nanofiber with a diameter of 215 nm is shown in Fig. 6. The three-point bending test was repeated for 50 cycles for the same nanofiber. The Young’s modulus value of the nanofiber fluctuates slightly because such a process cannot be exactly controlled at the same point every time. In general, after 50 cycles, PU nanofiber exhibits good durability with no significant degradation in Young’s modulus.

## Conclusions

In summary, the Young’s modulus of a single PU nanofiber prepared from electrospun method was measured by three-point bending test. The increasing Young’s modulus with decreasing diameter can be ascribed to the surface effect. Besides, the Young’s modulus decreases linearly with the increase in temperature in the range of 25 °C~60 °C. PU nanofiber exhibits good durability with no significant degradation in Young’s modulus even after 50 cycles.

## Abbreviations

1D: One-dimensional
AFM: Atomic force microscopy
DMF: N, N-dimethylformamide
PU: Polyurethane
SEM: Scanning electron microscopy
TGA/DSC: Thermogravimetric differential scanning calorimetry (TG/DSC)
THF: Tetrahydrofuran
